# Pollutant composition modification of the effect of air pollution on progression of coronary artery calcium: The Multi-Ethnic Study of Atherosclerosis

**DOI:** 10.1097/EE9.0000000000000024

**Published:** 2018-09-12

**Authors:** Joshua P. Keller, Timothy V. Larson, Elena Austin, R. Graham Barr, Lianne Sheppard, Sverre Vedal, Joel D. Kaufman, Adam A. Szpiro

**Affiliations:** a Department of Biostatistics, Johns Hopkins Bloomberg School of Public Health, Baltimore, MD; b Department of Civil and Environmental Engineering, University of Washington, Seattle, WA; c Department of Environmental and Occupational Health Sciences, University of Washington, Seattle, WA; d Departments of Medicine and Epidemiology, Columbia University, New York, NY; e Departments of Environmental and Occupational Health Sciences and Biostatistics, University of Washington, Seattle, WA; f Department of Biostatistics, University of Washington, Seattle, WA.

## Abstract

**Background:**

Differences in traffic-related air pollution (TRAP) composition may cause heterogeneity in associations between air pollution exposure and cardiovascular health outcomes. Clustering multipollutant measurements allows investigation of effect modification by TRAP profiles.

**Methods:**

We measured TRAP components with fixed-site and on-road instruments for two 2-week periods in Baltimore, Maryland. We created representative TRAP profiles for cold and warm seasons using predictive *k*-means clustering. We predicted cluster membership for 1005 participants in the Multi-Ethnic Study of Atherosclerosis and Air Pollution with follow-up between 2000 and 2012. We estimated cluster-specific relationships between coronary artery calcification (CAC) progression and long-term exposure to fine particulate matter (PM_2.5_) and oxides of nitrogen (NO_X_).

**Results:**

We identified two clusters in the cold season, notable for higher ratios of gases and ultrafine particles, respectively. A 5-μg/m^3^ difference in PM_2.5_ was associated with 17.0 (95% confidence interval [CI] = 7.2, 26.7) and 42.6 (95% CI = 25.7, 59.4) Agatston units/year CAC progression among participants in clusters 1 and 2, respectively (effect modification *P* = 0.006). A 40 ppb difference in NO_X_ was associated with 22.2 (95% CI = 7.7, 36.7) and 41.9 (95% CI = 23.7, 60.2) Agatston units/year CAC progression in clusters 1 and 2, respectively (*P* = 0.08). Similar trends occurred using clusters identified from warm season measurements. Clusters correlated highly with baseline pollution level.

**Conclusions:**

Clustering TRAP measurements identified spatial differences in composition. We found evidence of greater CAC progression rates per unit PM_2.5_ exposures among people living in areas characterized by high ratios of ultrafine particle counts relative to NO_X_ concentrations.

**What this study adds**

This article presents a novel investigation of how differences in traffic-related air pollution may modify the relationship between long-term particulate matter exposure and cardiovascular health. A distinctive feature is the use of near- and on-road pollutant measurements to inform predictions of pollution profiles using modern statistical clustering methods. This study provides evidence of greater health impact of exposure to pollution, with higher proportions of ultrafine particles within metropolitan regions.

## Introduction

The relationship between exposure to traffic-related air pollution (TRAP) and cardiovascular morbidity and mortality has been well established.^1–6^ TRAP is a complex mixture of many different particulate and gaseous components that can vary across fine spatial scales^7^ and is of particular interest in urban areas^1^. Our focus in this study is on directly emitted TRAP whose composition and concentration levels vary across an urban area with traffic intensity,^8^ distance to roadway,^7^ fuel type (heavy duty diesel versus gasoline), age and condition of vehicle, and source^9^ (tailpipe, brake wear, tire wear, etc.). There is a recognized need to move beyond single-pollutant epidemiological analyses and consider the effects of exposure to mixtures of pollutants.^10–14^

Complex multipollutant datasets are often analyzed using dimension reduction techniques, which simplify the complex variability of the data into a smaller set of features. Clustering methods, which provide a promising approach for understanding multipollutant health effects,^10^ partition multipollutant observations into a prespecified number of groups or clusters. This provides a categorical division of the data based on pollutant profile that simplifies the interpretation of simultaneous exposure to multiple pollutants.^15,16^ In the popular “*k*-means” algorithm, clusters are selected to minimize the (Euclidean) distance between each observation and the center, or representative exposure vector, of its assigned cluster.^17^ For analyses of administrative data, records can be assigned to a cluster based on city.^16,18^ For cohort studies, a classification model can be used to predict cluster membership at subject residences.^15^ Cluster membership for each subject or record can then serve as an effect modifier for an association between a single exposure and outcome.^10,15,16^ This allows for heterogeneity in the association between a single composite pollutant (e.g., NO_X_, fine particulate matter [PM_2.5_]) and health outcomes to be identified across groups distinguished by predicted differences in pollution composition at subject locations.

Prior modeling of spatial variation in TRAP across cities has focused on separate land-use regression models for ultrafine particles^19–22^ and volatile organic compounds (VOCs).^23^ While these models can provide estimates of associations with differences in level of a single TRAP component, the single-component approach cannot capture the mixture features identifiable from considering variation in multiple TRAP components simultaneously as can be done through clustering.

The Multi-Ethnic Study of Atherosclerosis and Air Pollution (MESA Air) investigated the association between long-term air pollution exposure and progression of subclinical measures of atherosclerosis, including coronary artery calcification (CAC).^24^ Using spatiotemporal predictions of individual pollutants, Kaufman et al.^6^ found an association between CAC progression and higher levels of NO_X_ and PM_2.5_ exposure. Recent supplemental monitoring campaigns have obtained an expansive suite of multipollutant measurements in MESA Air cities.^25,26^ These observations provide the opportunity to explore variation in CAC progression due to differences in TRAP composition.

## Methods

### Study population and exposure assessment

The MESA and MESA Air cohorts have been described extensively previously.^6,24,27^ In Baltimore, Maryland, 1081 participants were recruited between July 2000 and August 2002. Subjects received CT scans at baseline and at multiple follow-up visits through 2012. Scanner type varied by visit, with most baseline scans made using a Aquilion scanner (Toshiba) and later follow-up exams using a Volume Zoom scanner (Siemens). Scans were scored for coronary artery calcium using the Agatston method.^28^ Baseline characteristics of the cohort are summarized in Table 1 for the 1005 participants for whom complete covariate and exposure information was available. The study protocol was approved by the institutional review board at the coordinating and local study centers, and participants provided written informed consent.

**Table 1:**
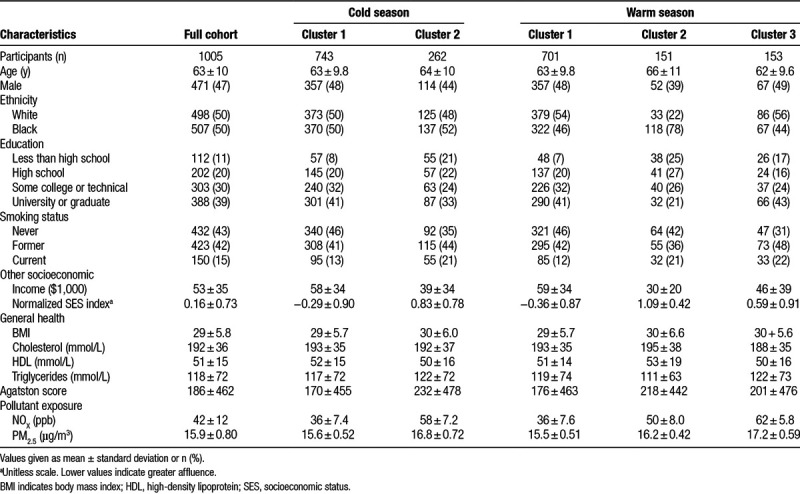
Baseline characteristics of the study population together and stratified by predicted membership in cold and warm season clusters

Predictions of participant-specific outdoor ambient exposures of NO_X_ and PM_2.5_ were made using a standardized set of spatiotemporal prediction models developed for MESA Air.^29^ These predictions were resolved to the exact residential addresses of participants at a 2-week time scale. Cross-validated R^2^ for the models indicated good out-of-sample prediction accuracy (R^2^ of 0.92 and 0.84, for NO_X_ and PM_2.5_, respectively).^29^ For modeling CAC, NO_X_ and PM_2.5_ exposure predictions were aggregated into long-term averages from recruitment through exam visit, based on participant residential history.

### Multipollutant TRAP data

Pollution measurements were made at 43 intersections within the Baltimore, Maryland, metropolitan area during 2-week periods in February 2012 and June 2012, which we refer to as the cold and warm seasons, respectively. Measurements were made in two seasons to capture differences in TRAP composition due to changes in sources and meteorology throughout the year. Most of the intersections were in residential areas and not on major roads, given the goal to characterize TRAP profiles relevant to subjects’ ambient exposures. Measurements of nitrogen dioxide (NO_2_), oxides of nitrogen (NO_X_), ozone (O_3_), and specific VOCs were made using stationary badge monitors (see Table 2). Carbon monoxide (CO) concentrations and particle number (PN) counts for different-size bins were measured using an on-road mobile platform at a collection of locations in and near the intersection by traversing the blocks bordering the intersection of interest. The smallest-size bin for PN counts captured particles 25 to 400 nm in diameter. Riley et al.^26^ provide a detailed description of the sample collection procedures. The mobile measurements were made during the afternoon commuting period and were adjusted for day-to-day variability by subtracting the fifth percentile of each pollutant and taking the median value of all adjusted observations at each location.^26^

**Table 2:**
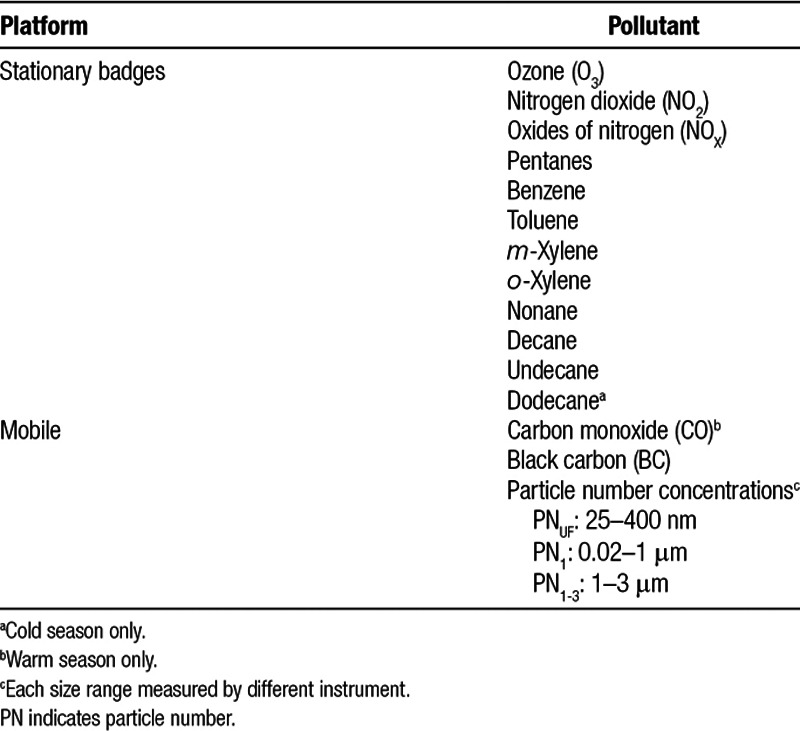
Pollutants measured on mobile and fixed monitor platforms

We scaled observations from both the badge and mobile platforms by the measured badge NO_X_ concentration at the respective location. Standardization by NO_X_ level allows clusters derived from the data to be informed by relative pollution composition and not solely by overall level. We then standardized these pollution fractions to have mean zero and unit standard deviation.

### Clustering

Using the predictive *k*-means method,^15^ we clustered the component species fractions separately by season. The predictive *k*-means method identifies cluster centers by simultaneously optimizing the deviation of the multipollutant observation from its assigned cluster center and the assignment of each monitor location to a cluster based on prediction variables, which are typically a function of spatial location. This results in clusters whose membership can be better predicted at subject locations compared to clusters from traditional *k*-means clustering, which does not incorporate prediction variables when identifying cluster centers. The predictive *k*-means method is implemented in the publicly available R package “predkmeans,” and additional technical details are provided in eAppendix 1; http://links.lww.com/EE/A17.

We used a large collection of geographic variables containing land use information, distance to roadways, emissions inventories, and other values derived from Geographic Information Systems (GIS). These covariates, listed in eTable 1; http://links.lww.com/EE/A17, have been used previously to develop spatial and spatiotemporal prediction models.^29–32^ We reduced the values of these geographic variables at monitor locations to a small set of principal component analysis (PCA) scores, which we included as prediction variables for modeling cluster assignment predictive *k*-means.

We chose the number of clusters and the number of PCA scores by 10-fold cross-validation (CV). We limited the models to between 2 and 5 clusters and 1 and 6 PCA scores. Models were compared according to their mean squared prediction error (MSPE), which is the sum of the squared distances between the observed pollutant fractions (after standardization) and their predicted cluster centers.^15^ This provides an aggregate estimate of the error in using predicted cluster membership relative to the observed value for each measured TRAP component. eAppendix 2; http://links.lww.com/EE/A17 provides additional detail for calculating this CV metric.

We predicted cluster membership at each participant residence using a multinomial logistic regression model (see eAppendix 1; http://links.lww.com/EE/A17). The covariates for prediction were PCA scores evaluated using geographic covariates at participant residence locations and based upon the relationship between variables and scores at monitor locations. We assigned cluster membership for each participant record according to residence at the time of the exam.

### CAC progression analyses

Following the approach of the primary MESA Air analyses,^6^ we estimated the association between CAC progression and pollution exposure (PM_2.5_ or NO_X_) via a mixed model. The model includes a cross-sectional component that models baseline CAC, a longitudinal component that accommodates time-varying confounders, and a time-varying component that includes transient factors affecting measurements.^6,33^ Variables included are age, sex, race/ethnicity, site, scanner type, adiposity, physical activity level, smoking and second-hand smoke exposure status, employment outside the home, total cholesterol level, high-density lipoprotein level, triglyceride level, statin use, an index of neighborhood socioeconomic status,^34^ education, and income. We excluded all data from participants after a coronary revascularization procedure.

We included cluster membership as an effect modifier for the longitudinal associations between CAC and PM_2.5_ (or NO_X_). In total, we fit four separate models, corresponding to the two pollutants of interest and the two groupings of the cohort based on the cold- and warm-season clusters. The coefficients from the cluster–pollutant–time interaction provide cluster-specific estimates of the association between pollution exposure (PM_2.5_ or NO_X_) and CAC progression. Statistical significance of the effect modification was assessed using a likelihood ratio test.

### Sensitivity analyses

Because we found strong correlation between membership in the identified clusters and baseline exposure levels for participants, we conducted sensitivity analyses that estimated cluster-specific CAC progression associations for alternative cluster definitions. To compare against clusters not derived from the TRAP measurements, we split the cohort into those with baseline pollution exposure (NO_X_ or PM_2.5_) above and below the cohort-wide median level. As a second sensitivity analysis, we orthogonalized the GIS covariates against baseline NO_X_ level (using year 2000 annual averages for monitor locations) and then computed new PCA scores from these modified covariates and clustered the pollutant measurements via predictive *k*-means. This sensitivity analysis was designed to identify clusters that were less correlated with the regional trend in baseline exposure levels.

## Results

### TRAP data

**Figure 1.**
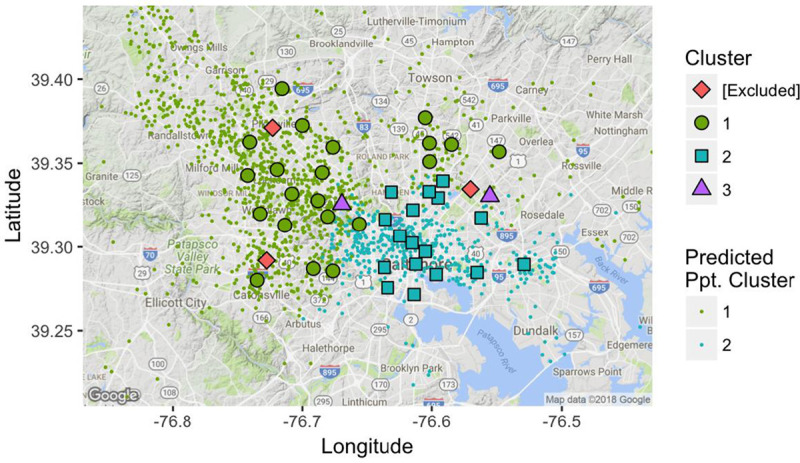
Monitoring locations, colored by membership in cold-season cluster.

**Figure 2.**
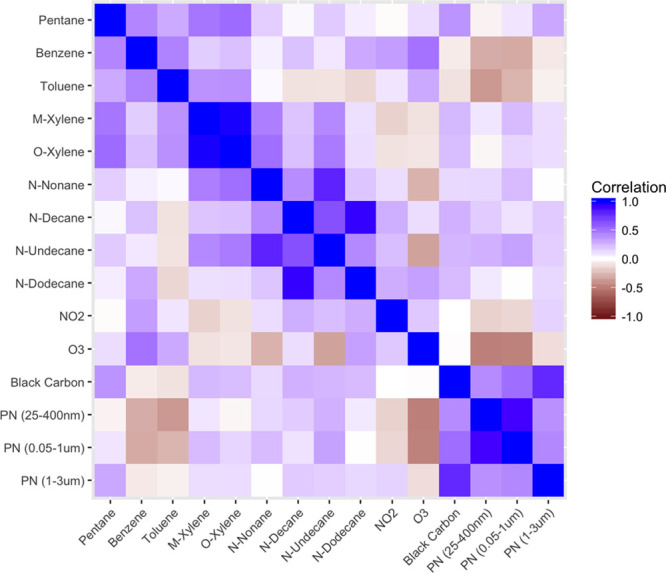
Heatmap of the correlation between measurements in the cold season. NO_2_ indicates nitrogen dioxide; O_3_, ozone; PN, particle number.

The locations of the 43 monitoring sites are provided in Figure 1. Three (different) locations in each of the cold and warm seasons were removed due to instrument error in the processing of badge measurements, leaving 40 sites for each season. Figure 2 shows a heatmap of the correlations between the pollutants in the cold season, after scaling by NO_X_. eFigure 1; http://links.lww.com/EE/A17 provides the analogous plot for the warm-season data.

### Clustering results

In the cold season, the predictive *k*-means model with three clusters and two PCA scores performed the best in CV (MSPE=16.82; eTable 1; http://links.lww.com/EE/A17). However, parameters in this model were not fully identifiable because one cluster included only two locations but had three coefficients for classification. Therefore, we chose the model with three clusters and one PCA score for the cold season because it was not overdetermined and had the second-best CV performance (MSPE = 16.94). In the warm season, the best CV results were for the model with three clusters and two PCA scores (MSPE = 15.32).

**Figure 3.**
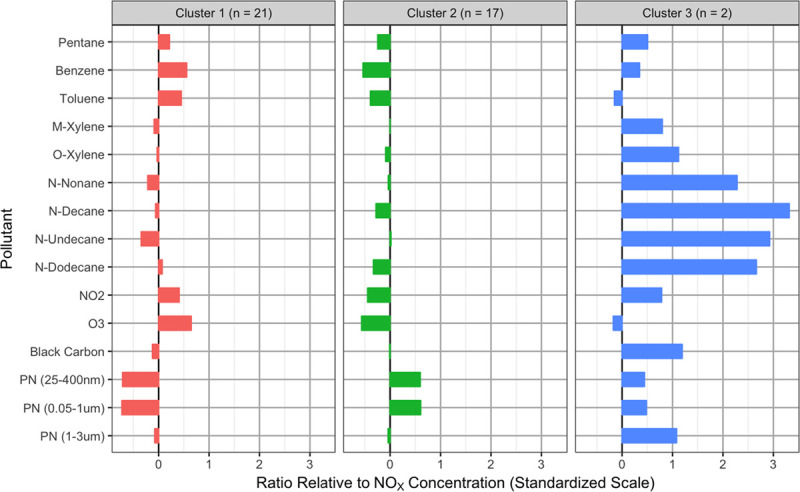
Cold-season cluster centers. NO_2_ indicates nitrogen dioxide; NO_X_, oxides of nitrogen; O_3_, ozone; PN, particle number.

The cluster centers from the cold season are depicted in Figure 3. Most monitors were assigned to Cluster 1 (21 locations) or Cluster 2 (17 locations). Cluster 1 was characterized by above average ratios of NO_2_ and ozone relative to NO_X_, while the profile for Cluster 2 showed the opposite trend, with lower fractions of gases but higher ratios of ultrafine (25–400 nm) and accumulation mode (0.05–1 μm) particle counts relative to NO_X_. Locations west and north of downtown were primarily assigned to Cluster 1, while those assigned to Cluster 2 were located closer to downtown (see Figure 1). Cold-season Cluster 3, which comprised two locations, had high ratios of almost all gases and particle sizes. A summary of cluster attributes is provided in Table 3.

**Table 3:**
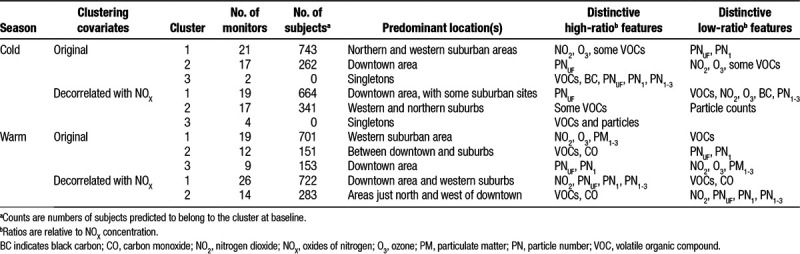
Descriptive summary of clusters

**Figure 4.**
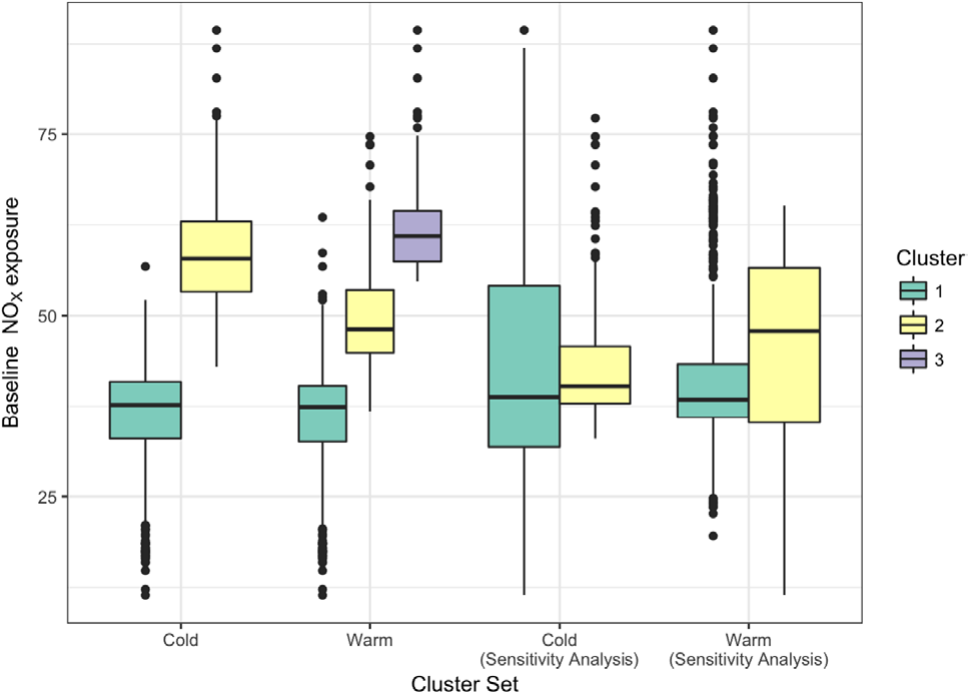
Baseline NO_X_ exposure by cluster membership. NO_X_ indicates oxides of nitrogen.

We predicted that, at baseline, 743 and 262 participants belonged to cold-season Clusters 1 and 2, respectively, and none to Cluster 3. Table 1 summarizes baseline characteristics of the study cohort, stratified by cold-season and warm-season cluster membership. In addition to the clear geographic patterns between clusters, participants in Cluster 1 tended to have higher levels of education and socioeconomic status than those in Cluster 2, while baseline NO_X_ and PM_2.5_ exposure levels were higher in Cluster 2 (Figure 4).

The centers for the clusters identified in the warm season are depicted in eFigure 2; http://links.lww.com/EE/A17. The first cluster (to which 19 locations were assigned) was characterized by lower ratios of VOCs and higher ratios of NO_2_, O_3_, and PN_1-3_. The second cluster (12 locations) had the highest ratios of all VOCs and CO but low ratios of particle counts. The third cluster (9 locations) was notable for its low PN_1-3_ counts and NO_2_ and O_3_ concentrations.

Warm-season cluster membership also showed a spatial pattern, with monitors located in the urban center primarily assigned to Cluster 1 and locations furthest from downtown generally assigned to Cluster 3 (eFigure 3; http://links.lww.com/EE/A17). Most participants (701 at baseline) were predicted to belong to Cluster 1 in the warm season. A total of 151 and 153 participants were predicted to belong to Clusters 2 and 3, respectively. Participants in Cluster 2 tended to have lower levels of income, education, and neighborhood socioeconomic status (Table 1). The warm-season clusters also showed patterns in baseline NO_X_ and PM_2.5_ concentrations at residences (Figure 4).

In the sensitivity analysis using clusters based on GIS covariates with correlation with baseline NO_x_ removed, the best models (according to CV MSPE) had three clusters and two PCA scores in the cold season and two clusters and four PCA scores in the warm season (eTable 1; http://links.lww.com/EE/A17). Similar to cold-season Cluster 1 from the primary analysis, cold-season Cluster 1 from the sensitivity analysis had below-average ratios for all TRAP components relative to NO_X_ except for ultrafine and accumulation mode particle counts (eFigure 4; http://links.lww.com/EE/A17). However, this cluster included several monitors from suburban areas in addition to those located in the downtown area (eFigure 5; http://links.lww.com/EE/A17). Cold-season Cluster 2 from the sensitivity analysis had low ratios of ultrafine and accumulation mode particle counts, similar to Cluster 1 from the primary analysis. Cluster 3 had high ratios of almost all TRAP components relative to NO_X_ but only included four monitors. In the warm season, Cluster 1 broadly resembled a combination of Cluster 1 and 3 from the primary analysis and had high particle count fractions and low VOC fractions, while Cluster 2 showed the opposite trend (eFigure 6; http://links.lww.com/EE/A17). In both seasons, the baseline NO_X_ exposures for participants did not show a strong correlation with cluster membership (Figure 4).

### CAC progression estimates

In a model without effect modification by cluster, a difference of 5 μg/m^3^ in PM_2.5_ was associated with 23.0 Agatston units per year CAC progression (95% confidence interval [CI] = 14.2, 31.7). When cold-season cluster membership was used as an effect modifier, the estimated association with a 5 μg/m^3^ difference in PM_2.5_ was 17.0 (95% CI = 7.2, 26.7) units/year for participants belonging to Cluster 1 and 42.6 (95% CI = 25.7, 59.4) units/year for participants in Cluster 2 (see Table 4). The model with cluster-specific progression terms was significantly different from the city-wide model without cluster interactions (*P* = 0.006). Effect modification by warm season cluster was not statistically significant (*P* = 0.10) but showed a similar trend: 17.1 (95% CI = 7.1, 27.0), 24.7 (95% CI = –0.1, 49.6), and 39.8 (95% CI = 20.0, 59.6) Agatston units/year CAC progression associated with 5 μg/m^3^ difference in PM_2.5_ among participants in Clusters 1, 2, and 3, respectively. The median baseline PM_2.5_ concentration was 15.77 μg/m^3^. The estimated associations (per 5 μg/m^3^ difference in PM_2.5_) were 13.7 (95% CI = 2.1, 25.4) and 33.1 (95% CI = 21.2, 44.9) units/year for participants with baseline exposure below and above this value, respectively.

**Table 4:**
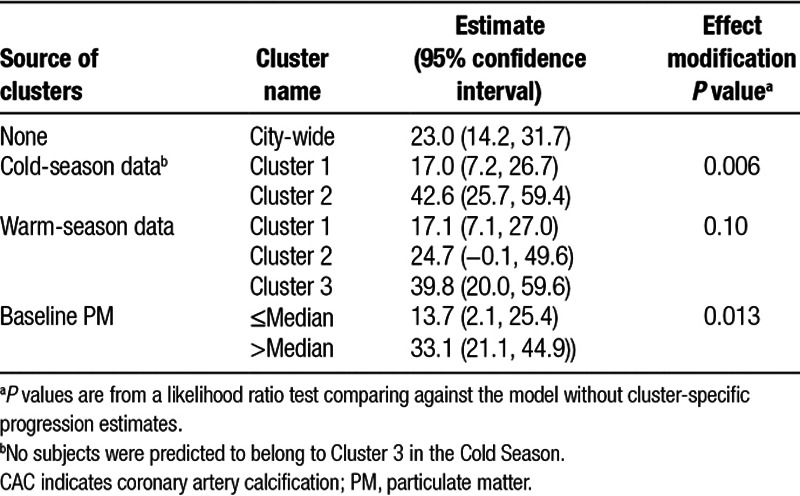
Cluster-specific estimates of the association between CAC progression, in Agatston units per year, and differences of 5 μg/m^3^ PM_2.5_

The estimated associations with NO_X_ exposure showed a similar trend across clusters (see Table 5). A difference of 40 ppb in NO_X_ exposure was associated with 22.2 (95% CI = 7.7, 36.7) and 41.9 (95% CI = 23.7, 60.2) Agatston units/year CAC progression among participants in cold-season Clusters 1 and 2, respectively. These results were not significantly different (*P* = 0.076) from the estimate for all participants pooled together (28.2 units/year, 95% CI = 17.1, 39.3). Estimates for the warm-season clusters followed the same pattern as for PM_2.5_: 20.7 (5.9, 35.6), 25.7 (−2.0, 53.4), and 38.1 (16.8, 59.5) for Clusters 1, 2, and 3, respectively. There was no evidence for effect modification by baseline NO_X_ (*P* = 0.64), although the point estimates had a similar trend to results from the model with effect modification by cold-season cluster.

**Table 5:**
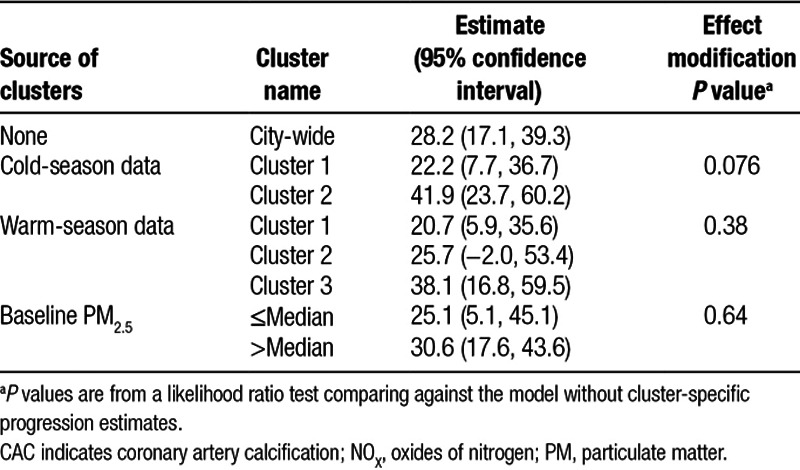
Cluster-specific estimates of the association between CAC progression, in Agatston units per year, and differences of 40 ppb NO_X_.

In the sensitivity analysis using covariates that had been orthogonalized with respect to NO_X_, there was little difference in the estimated association between pollution exposure and CAC progression between clusters from the warm season (eTable 2; http://links.lww.com/EE/A17). When clustering subjects using the cold-season data, the estimates from Cluster 1 vs. 2 were significantly different (*P* = 0.023 and *P* = 0.017 for PM_2.5_ and NO_X_, respectively). The confidence intervals for the progression estimates in Cluster 2 were notably broad and included zero for NO_X_ and PM_2.5_. In cold-season Cluster 1, the estimated association between CAC progression and both PM_2.5_ and NO_X_ exposure was similar to, but greater than, the estimates from the city-wide analysis without clustering. Similar to the trend observed in the original analysis, the largest associations with CAC progression were observed among people predicted to be exposed to pollution with higher ratios of ultrafine and accumulation-mode particles relative to NO_X_.

## Discussion

We have used a novel collection of near- and on-road multipollutant measurements to assess how spatial differences in pollution composition affect the relationship of PM_2.5_ and NO_X_ exposure with CAC. By reducing dimension of the measurements using clustering and categorizing the cohort by these clusters, we were able to incorporate multipollutant information into a longitudinal model for CAC progression.

In our primary analysis, we found significant differences in the association between CAC progression and PM_2.5_ exposure when grouping subjects by predicted cold-season TRAP profile. The association was strongest among participants in Cluster 2, which was notable for its locations being primarily in the downtown area and its above average ratios of ultrafine and accumulation-mode particle counts relative to NO_X_. Freshly emitted vehicle exhaust is one likely source for these higher particle counts in the urban center.^9^ When grouping by predicted warm season TRAP profiles, the estimated association between CAC progression and PM_2.5_ exposure was weaker in the cluster with lower ratios of ultrafine particle counts (Cluster 2) and strongest in the cluster with high ratios of ultrafine particle counts (Cluster 3).

Our results suggest that overall PM_2.5_ exposure among individuals whose ambient residential TRAP concentrations have high levels of ultrafine particles relative to NO_X_ has greater adverse cardiovascular impacts than exposure among participants whose ambient residential TRAP concentrations have different composition. Evidence for increased risk of atherosclerosis due to exposure to ultrafine particles, relative to larger particle sizes, has been found in mice^35^. Ultrafine particles have also been linked to a broader set of inflammation- and endothelial-related outcomes.^36,37^ Although a defining feature of the identified TRAP profiles were the relative fractions of ultrafine particle counts, the moderately high correlation between ultrafine and accumulation-mode particle (Figure 2 and eFigure 1; http://links.lww.com/EE/A17) mean that the differences between clusters cannot be attributed to ultrafine particles alone. The lower fractions of VOCs and gases in cold-season Cluster 2 and warm-season Cluster 1 may also play a role in the observed differences.

A striking feature of the clustering results is the strong correlation between cluster membership, geographic location, and overall PM_2.5_ and NO_X_ levels. The predictive *k*-means clustering procedure incorporated an aggregation of geographic covariates via the PCA scores, which have a strong gradient from downtown out to the suburbs. These covariates are similar to those used for predicting exposures in the MESA Air cohort,^29^ which may drive some of the correlation between cluster membership and exposure level. This correlation makes it difficult to determine whether the between-cluster differences identified are due to differences in TRAP composition or differences in baseline exposure. We addressed this concern by conducting a sensitivity analysis that removed variability from the covariates that is explainable by baseline NO_X_ and yielded clusters that did not correlate well with baseline exposure. Using the cold-season TRAP measurements, the sensitivity analysis found larger associations between CAC progression and PM_2.5_ exposure among participants predicted to have TRAP profiles with higher fractions of ultrafine particles (Cluster 1). This supports the primary results, which found greater rates of progression among people predicted to have TRAP profiles with higher fractions of ultrafine particles.

Predicted cluster membership correlated with socioeconomic status, in addition to baseline exposure and geographic location. Strong relationships between socioeconomic status and pollution exposure have been previously identified in this cohort.^38^ The results from our analysis, however, identify differences that are not fully explainable by socioeconomic differences alone. In our primary analyses, participants in cold-season Cluster 1 and warm-season Cluster 1 had higher levels of affluence and weaker, but still non-zero, estimated associations between CAC progression and PM_2.5_ and NO_X_ exposure. However, participants in warm-season Cluster 2 had the lowest income and neighborhood-level socioeconomic index, but their estimated association between PM_2.5_ and NO_X_ exposure and CAC progression was very similar to the city-wide average. Additionally, participants in warm-season Cluster 3 had higher average income and were more affluent but also had a stronger estimated association between PM_2.5_ and CAC progression than participants in Cluster 2.

We estimated TRAP profiles for the cold and warm season separately and found similar trends in the relative component fractions across seasons. This could be due to similar widespread sources of directly emitted TRAP in each location across seasons, in contrast to residential heating sources or secondary pollutants that vary by season. The trend of effect modification by predicted profile was similar as well, although with different levels of statistical evidence. This is likely due in part to the overlap between the predicted cluster membership.

A limitation of this analysis was the relatively small number of locations at which component measurements were made. Although a sample size of 40 locations is far more than the one or two locations per city at which component data is typically available via the Chemical Speciation Network, it is nonetheless a relatively small sample for building a cluster prediction model. Including spatial splines in the prediction model as a mechanism for spatial smoothing is impractical with cluster sizes of 21 and 17 from the cold-season data. This leads to the prediction model being derived from one or two PCA scores, which can capture small-scale variability but can also be dominated by larger trends, as was the case in this study. The results may be impacted by differences in the time period of the data. The geographic variables used for cluster prediction were from the period 2000–2006. The cluster profiles were derived from multipollutant measurements in 2012 but were used to predict representative exposure profiles for the entire study period. It is possible the TRAP profiles and the relationship between geographic covariates and TRAP within the Baltimore region changed over time, although major highway patterns and industrial sources were largely stable.

The city-wide and cluster-specific estimates were all greater than the estimated association in the full MESA Air cohort, which includes participants from five other metropolitan areas. In the full cohort, differences of 5 μg/m^3^ difference in PM_2.5_ and 40 ppb in NO_X_ were associated with 4.1 (95% CI = 1.4, 6.8) and 4.8 (95% CI = 0.9, 8.7) Agatston units/year CAC progression, respectively.^6^ While the exposure prediction models for Baltimore did have the best overall predictive accuracy,^29^ the difference in progression estimates is not likely due to exposure assessment accuracy alone. Differences in pollution composition between the six MESA Air cities could potentially cause some of this difference; however, such differences are masked by between-city differences in cohort members. The defining feature of the MESA cohort is the overrepresentation of different ethnicities. The Baltimore sub-cohort, however, only includes white and black participants, while the other cities also have different racial-ethnic groups, which could be one source of the differences from the Baltimore-only results.

We have presented a novel approach to using multipollutant TRAP measurements within a metropolitan area to assess effect modification by pollution composition in longitudinal relationships between pollution exposure and CAC. Our results found that the same difference in PM_2.5_ or NO_X_ concentration was associated with faster CAC progression among participants living in areas predicted to have higher ratios of ultrafine particle counts relative to NO_X_ during the cold season. These results highlight how incorporating multipollutant measurements into health effect analyses can yield insight into heterogeneity in the relationships between air pollution exposure and health.

## Conflict of interest statement

The authors declare that they have no conflicts of interest with regard to the content of this report.
